# Intraoperative assessment of surgical margins using “*en face*” frozen sections in the management of cutaneous carcinomas^☆^

**DOI:** 10.1016/j.abd.2021.09.013

**Published:** 2022-07-05

**Authors:** Ana Carolina Vasconcellos Guedes Otsuka, Eduardo Bertolli, Mariana Petaccia de Macedo, Clovis Antonio Lopes Pinto, João Pedreira Duprat Neto

**Affiliations:** a Plastic Surgeon, A.C. Camargo Cancer Center, São Paulo, SP, Brazil; b Department of Cutaneous Oncology, A. C. Camargo Cancer Center, São Paulo, SP, Brazil; c Department of Pathology, Hospital Sírio Libanês, São Paulo, SP, Brazil; d Department of Pathology, A. C. Camargo Cancer Center, São Paulo, SP, Brazil

**Keywords:** Keratinocytes, Paraffin, Skin neoplasms

## Abstract

**Background:**

Basal cell and squamous cell carcinomas (BCC and SCC) are the most common types of cancer worldwide. Intraoperative assessment of surgical margins by frozen section has been widely used to ensure disease-free margins. The intraoperative “*en face*” freezing technique evaluates all peripheral and deep margins.

**Objective:**

To report the results of the “*en face*” freezing technique in relation to tumor recurrence and agreement with paraffin-embedded tissue examination.

**Methods:**

Retrospective analysis of patients undergoing surgical excision of BCC and SCC at the A. C. Camargo Cancer Center, Brazil.

**Results:**

This study included 542 skin carcinomas, which were excised from 397 patients. A total of 201 male patients (50.6%), and 196 female patients (49.4%) were assessed, whose mean age was 64 years. The tumors were mostly located on the head and neck region (87.8%). BCC corresponded to 79.7% of the cases. The mean follow-up was 38 months. Tumor relapse occurred in 0.86% of the primary tumors and 3.7% of recurrent tumors. The result of the intraoperative “*en face*” frozen section evaluation was in agreement with the final result of the anatomopathological examination (paraffin test) in 98% of the lesions.

**Study limitations:**

Not having a minimum follow-up time of 5 years for all patients.

**Conclusion:**

The “*en face*” freezing technique shows low tumor relapse, being reliable and safe to guarantee negative surgical margins of the tumor.

## Introduction

The skin cancers basal cell carcinoma (BCC) and squamous cell carcinoma (SCC) are more prevalent than all other types of cancer combined.^1^, ^2^ In Brazil, they account for 30% of all malignant tumors registered in the country. According to data from the National Cancer Institute (INCA, Instituto Nacional de Cancer), 176,930 new cases were estimated in 2020, of which 83,770 in men and 93,160 in women.^3^

According to the NCCN® Guidelines, the primary goal of treatment for BCC and SCC is the complete surgical removal of the tumor and maximum preservation of function and aesthetics.^2^

An excision made with a wide surgical margin can lead to disfigurement and unnecessary scarring, while conservative resections can result in incomplete tumor removal and increased local recurrence, especially in lesions with a high risk of recurrence. The BCC and SCC high-risk characteristics are: depth of invasion (>6 mm or invasion beyond the subcutaneous tissue for SCC), poorly differentiated histopathological differentiation, high-risk anatomical location (face, ear, pre/post-auricular region, genitalia, hands, and feet), perineural involvement, recurrent lesions after surgery and/or radiotherapy, immunosuppression, and poorly-defined borders.^2^

The intraoperative assessment of surgical margins of the lesion using frozen section evaluation has been widely used to try to ensure disease-free margins, making it safer to plan the definitive reconstruction.^4^, ^5^ Mohs micrographic surgery (MMS) is one of the proposed techniques for high-risk BCC and SCC, as it allows intraoperative analysis of 100% of the excision margin.^2^ However, it is important to have an acceptable and safe alternative to MMS, as this technique can be financially unfeasible and have limited availability.^5^ Another technique where there is also a complete assessment of all margins is the intraoperative assessment of surgical margins using “*en face*” frozen sections, which is a possible alternative to MMS.^4^, ^5^, ^6^

The term “*en face*” is used to say that when examining the surgical specimen during intraoperative freezing, the analyzed margin sections are processed parallel to the surgical margin.^7^

The present study objective is to report the results of the “*en face*” freezing technique regarding tumor recurrence and agreement with paraffin sections, in the routine of an institution.

## Material and methods

A retrospective analysis was carried out of patients submitted to surgical excision of BCC and SCC tumors at the Skin Cancer Center of A. C. Camargo Cancer Center, São Paulo/SP – Brazil, between january and December of 2013. The local Research Ethics Committee approved the present study, under number 2602/18.

### Patients

Patients with BCC and/or SCC, primary or recurrent, who were submitted to the “*en face*” freezing technique, regardless of the lesion topography, were included in the study. The non-inclusion criteria were patients with other types of skin neoplasms and patients who did not undergo the intraoperative “*en face*” freezing technique.

The following clinical, epidemiological, anatomopathological, and treatment data of each patient were evaluated: sex, age, type and subtype of tumor, type of reconstruction, number of resections required to obtain tumor-free surgical margins, the result of the intraoperative examination of the “*en face*” freezing technique and the final anatomopathological result after conventional histopathological processing in paraffin blocks, need for complementary treatment, time of follow-up and tumor recurrence (recurrence was defined as the reappearance of the lesion in the clinical follow-up confirmed by histopathological examination). Primary lesions were considered as clinically recently diagnosed BCCs or SCCs, and secondary lesions as those that had been previously treated in other hospitals and recurred.

### Statistical analysis

Descriptive analysis was performed by evaluating the medical records. Simple logistic regressions were used to identify variables associated with the assessed outcomes. All variables with a p-value <0.05 were included in the multiple logistic regression models. The IBM SPSS v. 20.0® software was used for the statistical analysis.

### Intraoperative “*en face*” freezing technique

The “*en face*” freezing technique allows the examination of all tumor margins. All peripheral and deep margins of the specimen analyzed using the “*en face*” freezing technique were sent for conventional histopathological processing in paraffin blocks. Thus, the results of the intraoperative examination using the “*en face*” frozen sections were compared with the final anatomopathological result after histopathological processing in paraffin blocks.

**Figure 1:**
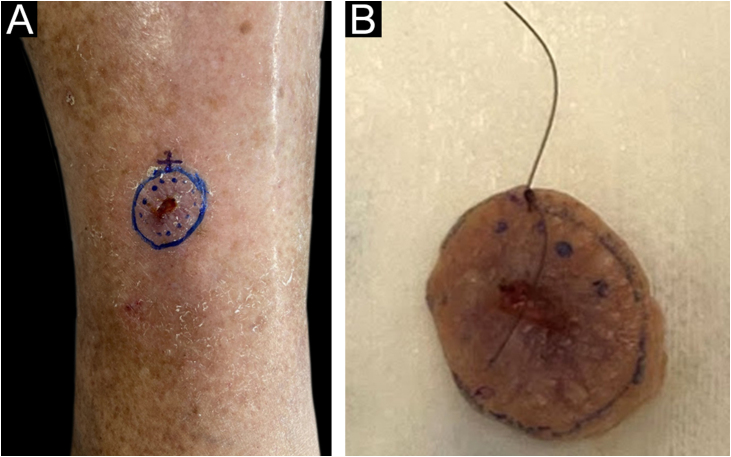
(A), Clinical aspect of the lesion before excision with dermoscopic delimitation of the surgical margins. (B), Sample resected and identified with surgical thread by the surgeon; reference point at the 12 o’clock position.

The surgical procedure was performed as follows. The skin tumor resection margins were identified with the aid of a manual dermoscope used by the surgeon responsible for the procedure, with the objective of achieving free resection margins. It was not possible to document the surgical margin used for excision, as this was a retrospective study. The excision was performed under general anesthesia, local anesthesia plus sedation, pure local anesthesia, or spinal anesthesia plus sedation, depending on the patient’s comorbidities, reconstruction planning, and lesion size. Lesion excision was performed with a straight circumferential border and a deep flat margin, different from the angled incision of the MMS resection. Fig. 1 shows the clinical appearance of the lesion before excision with the dermoscopic delimitation of the surgical margins.

**Figure 2:**
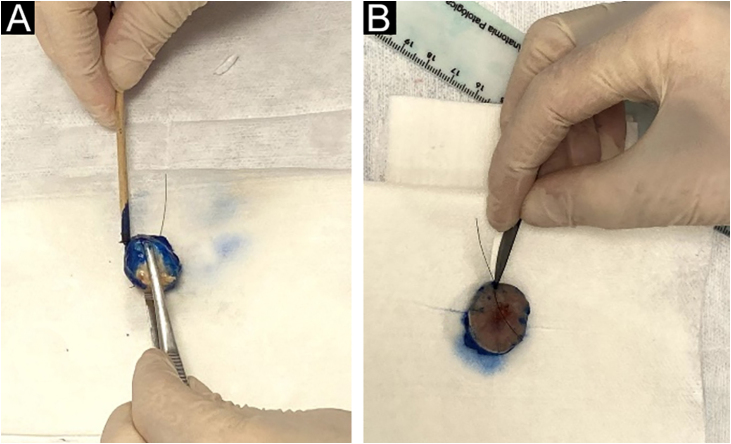
(A), The peripheral and deep margins of the specimen are painted by the pathologist. (B), Excision of the peripheral margin of the specimen, to be sent for the preparation of the frozen sections, consists of a 2.0 to 3.0 mm thick specimen obtained at the reference point with an incision parallel to the peripheral margin.

The intraoperative examination to assess the surgical margins of the excised lesion begins when the pathologist receives the fresh specimen with prior identification by the surgeon of the 12 o’clock patient reference point. All lateral and deep margins of the specimen are then painted with Indian ink, as shown in Fig. 2.

**Figure 3:**
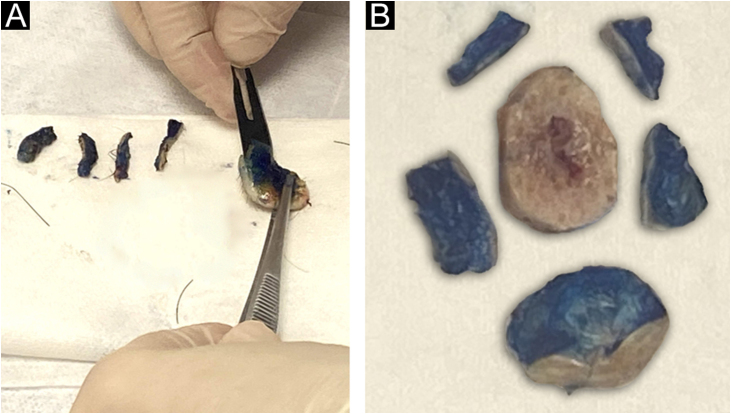
(A), Deep margin excision consisting of a parallel section containing the inked surface margins. (B), Schematic view of the final result of the circumferential margins separated into sectors and the deep margin.

The lateral margins of the specimen are sectioned into 4 sectors considering the 12 o’clock fiducial point previously identified by the surgeon and in sections considering the clockwise direction of the specimen as follows: 12 to 3 o’clock, 3 to 6 o’clock, 6 to 9 o’clock and 9 to 12 o’clock (“clock face” orientation method). These margins are separated from the specimen using an incision with the aid of a surgical blade at the starting point (e.g., 12 o’clock) and an internal resection 2.0 to 3.0-mm thick in the skin parallel to the circumferential border. The entire lateral extension of that sector is considered for microscopic evaluation. The deep margin is analyzed in a parallel section, tangential to the surgical margin. The procedure is illustrated in Fig. 3.

The fragments representative of each margin are then individually and sequentially submitted to frozen sections by freezing them in embedding medium (tissue tek®) and freezing at −30 °C in the cryotome, with the ink side (or external side) facing the cutting face of the device. Sections measuring 5.0 micrometers are obtained, followed by staining with hematoxylin & eosin, mounting of slides, and evaluation by the pathologist.

The margins are reported by the pathologist separately according to the identification of the sectors as neoplasm-free margins or neoplasm-compromised margins. When a sample of the fragment is positive for tumor cells after the histopathological examination, an additional tissue sample in the area affected by the tumor will be taken and sent again for analysis by the pathologist. The procedure is repeated until neoplasm-free margins are obtained, and then the surgeon performs the reconstruction of the affected area after lesion removal. All samples analyzed as frozen sections (peripheral and deep margins) undergo conventional histopathological processing in paraffin blocks to confirm the diagnosis of the frozen specimen performed intraoperatively.

## Results

### Overall results

The present study consisted of 542 skin carcinomas, excised from 397 patients (201 men and 196 women), with a mean age of 64 years. The clinical and pathological characteristics of the cases included in the study are shown in Table 1. The lesion size obtained by measuring the pathological specimen (cm) was on average 2.0 × 1.4 × 0.56 (largest diameter × smallest diameter × thickness).

**Table 1:** Qualitative characteristics of patients submitted to excision of basal cell carcinoma (BCC) and squamous cell carcinoma (SCC), from January to December 2013, at A. C. Camargo Cancer Center.

Variable	Categories	n	(%)
Tumor location	Scalp	28	5.2%
	Forehead	57	10.5%
	Nose	199	36.7%
	Lip	37	6.8%
	Malar region	47	8.7%
	Temporal region	34	6.3%
	Retroauricular region	3	0.6%
	Ear	33	6.1%
	Eyelid	25	4.6%
	Mento	4	0.7%
	Cervical region	9	1.7%
	Trunk	9	1.7%
	Dorsal region	5	0.9%
	Flank	1	0.2%
	Limbs	51	9.4%
Histopathological subtypes of cutaneous basal cell carcinoma	Nodular	104	29.4%
	Superficial	42	11.8%
	Sclerodermiform	33	9.3%
	Micronodular	9	2.5%
	Mixed^a^	159	45%
	Others^b^	5	1.3%
Type of anesthesia	Exclusively local	17	3.1%
	Local and sedation	192	35.4%
	General	329	60.7%
	Block	4	0.7%
Initial lesion presentation	Treatment naïve	463	85.4%
	Recurrent Tumor	79	14.6%
Perineural Invasion	Present	9	1.7%
	Absent	533	98.3%
First “*en face*” freezing sections	Free margins	392	72.3%
	Compromised margins^c^	150	27.7%
	No	5	0.9%
Complementary treatment (after surgery)	Radiotherapy	511	96.6%
	Complementary surgery	11	2.1%
	Topical treatment	2	0.4%

a More than one histopathological subtype.

b Other subtypes of BCC: adenoid and basosquamous tumor.

c In 116 lesions, intraoperative surgical margin one enlargement was required to obtain tumor-free margins. In 20 lesions, two enlargements were necessary. In 11 lesions, three enlargements were necessary. In two lesions, four enlargements were required and in one lesion, six surgical margin enlargements were required to obtain free margins.

The most common topography of the lesions was the head and neck region (87.8%), followed by extremities (9.4%) and the trunk (2.8%). The lesions affecting the extremities were: six lesions on the hands, three lesions on the feet, five on the pre-tibial region, thirteen on the upper limbs, and 24 on the lower limbs. BCC corresponded to 79.7% of the cases and SCC to 20.3%.

The mean follow-up was 38 months. A total of 53 cases were lost to follow-up. The maximum follow-up time was 74.43 months. The types of reconstruction after surgical excision of the skin carcinoma were: 454 local flaps (83.8%), 39 skin grafts (7.2%), 34 primary closures (6.3%), 14 cases of combined technique (2.6 %), and one microsurgical case (0.2%).

### Recurrence

Total tumor recurrence (median of 38 months) was 1.4%, being 0.86% for treatment-naïve lesions and 3.7% for secondary lesions.

The associations between the variables and recurrence-free survival were also evaluated through Cox regression, as shown in Table 2.

**Table 2:** Single and multiple Cox regression with variables associated with recurrence-free survival in patients submitted to BCC and SCC excision from january to december 2013, at A. C. Camargo Cancer Center.

Variable	Single COX regression			Multiple Cox regression		
	p	RR	95% CI	p	RR	95% CI
Perineural invasion	0.048	8,507	1,023‒70,758	0.02	13,049	1,504‒113,216
Number of enlargements until free margins were obtained	0.008	1,841	1,170‒2,895	0.005	1,872	1,205‒2,907

RR, relative risk; 95% CI, confidence interval; BCC, basal cell carcinoma; SCC, squamous cell carcinoma.

Using multiple Cox regression, the association with positive perineural invasion showed a 13-fold increase in the risk of having tumor recurrence. The number of intraoperative surgical margin enlargements was also associated, with a 1.8-fold increase in the risk of tumor recurrence for each margin enlargement. Hence, the greater the number of margin enlargements, the greater the risk of tumor recurrence in the future.

Of the 7 relapsed cases, 4 were BCCs. Of these, three were of mixed histopathological subtype (more than one histopathological subtype) and one was a superficial BCC, all located on the face (two lesions on the scalp, one on the forehead, and one on the nose), three were previously recurred tumors (mixed histopathological subtype) and had compromised margins in the first surgical resection, with a lesion being enlarged up to four times until attaining tumor-free surgical margins. The other three relapsed cases were SCCs, also located on the face (ear, scalp and nose), with all three cases being treatment-naïve lesions.

All patients who showed relapse had a minimum follow-up time of four years. The relapses occurred between five and 44 months after the surgical procedure. The median recurrence-free survival was 25 months (SD 5‒44).

The lesions included in the study were reviewed and 536 lesions were considered to be high risk. The total tumor relapse rate in high-risk lesions was 1.3%, with 0.87% relapse for primary tumors and 3.7% for recurrent tumors.

### Relationship between intraoperative “*en face*” freezing technique and the result of histopathological processing in paraffin blocks (concordant or discordant)

The results of the histopathological processing in paraffin blocks were in agreement with the results of intraoperative “*en face*” frozen sections in 98% of the lesions.

The associations between patient variables and the results of the permanent pathology (paraffin) were evaluated in relation to the agreement with the “*en face*” freezing technique. The qualitative variables described in Table 1 did not show any statistical significance. On the other hand, statistical significance was observed in relation to the quantitative variables.

A direct relationship was observed between the number of lesions operated on in the same patient and the increase in disagreement between diagnostic methods. Each lesion operated on in the same patient increases the risk of disagreement between the final anatomopathological result and the intraoperative “*en face*” frozen section by 24%. Regarding margin enlargements, for each lesion (margin) enlarged, there is a two-fold increase in the risk of having a disagreement between the final anatomopathological result and the intraoperative “*en face*” frozen section. The results are summarized in Table 3.

**Table 3:** Single and multiple logistic regression with variables associated with the final anatomopathological result (concordant and discordant) in patients submitted to BCC and SCC excision from january to december 2013, at A. C. Camargo Cancer Center.

Variable	Single logistic regression			Multiple logistic regression		
	p	OR	95% CI	p	OR	95% CI
Number of excised lesions	0.001	1,227	1,089‒1,383	0.000	1,243	1,101‒1,403
Number of enlargements until free margins were achieved	0.004	1,945	1,231‒3,073	0.005	2,035	1,237‒3,347

OR, odds ratio; 95% CI, confidence interval; BCC, basal cell carcinoma; SCC, squamous cell carcinoma.

All variables with p < 0.05 were included in the multiple logistic regression (Table 3).

## Discussion

A total of 397 patients were analyzed, with 542 skin carcinoma lesions (BCC and SCC), between january 1 and december 31, 2013 (a one-year period), submitted to intraoperative “*en face*” freezing technique. This is an important fact, as there are no studies in the literature evaluating the use of the “*en face*” intraoperative freezing technique with a similar number of cases in such a short period of time. Table 4 summarizes some important characteristics of studies found in the literature using the “*en face*” freezing technique.

**Table 4:** Data found in the literature on the use of intraoperative “*en face*” frozen sections.

Authors	Studied period	Number of lesions	Location	Follow-up (months)	Recurrence rate (%)
Gayre et al^10^	1985 to 2008	1638	Periocular BCC	84	1.7%
Wong et al.^4^	1992 to 2001	534	Periocular BCC	60	2.2%^a^
Gill et al.^13^	1997 to 2011	77	Periocular BCC	54	1.3%
Resti et al.^12^	1998 to 2010	110	Eyelid BCC	63	1.8%
Kvannli et al.^11^	1999 to 2007	262	Periorbital BCC and SCC	‒	‒
Menesi et al.^5^	2002 to 2006	53	Facial BCC	36	1.7%
Tullett et al.^7^	2003 to 2009	78	Periocular BCC	23	1%
Nizamoglu et al.^9^	2010 to 2014	70	High-risk carcinomas	12	0%
Present study^b^	2013	542	Head and neck, trunk and limb BCCs and SCCs	38	1.4%

BCC, basal cell carcinoma; SCC, squamous cell carcinoma.

a Primary lesion. ^b^ Current study.

Most studies found in the literature on the “*en face*” freezing technique are restricted to a specific topography, describing lesions on the periorbital region and on the face, but the present study had a broader inclusion criterion regarding location. This demonstrates that this freezing technique can be safely used in other anatomical sites.

The present study showed a total of 1.4% tumor recurrence (7 of 542 lesions); after stratifying this result, it showed 0.86% of recurrence for primary tumors and 3.7% for recurrent tumors, with a median follow-up of more than three years. All patients with tumor recurrence showed no disagreement between the result of the intraoperative “*en face*” freezing technique and the final result of the anatomopathological examination (paraffin processing). This demonstrates that this freezing technique is safe and was not the determining factor for tumor recurrence, which is more related to tumor biology.^4^, ^5^, ^8^ The overall tumor recurrence rate in the present study is similar to that of the literature.

**Figure 4:**
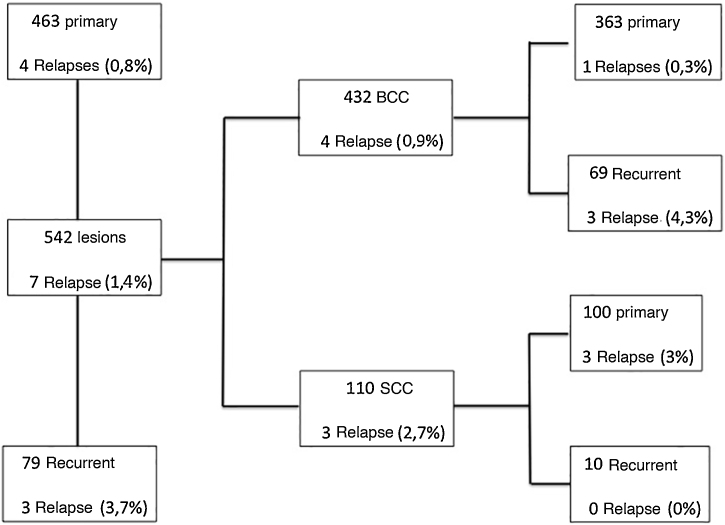
Flowchart specifying the results separately for BCC and SCC, regarding primary and recurrent tumors and the recurrence rate, which is shown as percentage.

Based on a literature review, the five-year recurrence rate for MMS was 0.6% to 3% for primary BCC and 6% to 10% for recurrent BCC.^9^, ^10^ A flowchart was created, specifying the results for BCC and SCC separately, regarding primary and recurrent tumors and recurrence rate, as shown in Fig. 4. Therefore, the recurrence rate for primary BCC (0.3%) and recurrent BCC (4, 3%) in the present study, using the intraoperative “*en face*” freezing technique, was similar to the MMS results reported in the literature. However, one cannot compare the results of the “*en face*” freezing technique with MMS, as there are no studies in the literature comparing these two techniques.

The authors did not report the surgical time observed with the use of the “*en face*” freezing technique, as discrepancies were found among the notes in the medical files, making the data uncertain; therefore, they chose not to use it. Tullett et al., report that the time for the preparation of slides when using the “*en face*” freezing technique is 45 to 75 minutes, showing that this freezing technique is a fast one.^8^

All peripheral and deep margins analyzed in the frozen sections were sent for analysis in paraffin blocks, aiming to review the frozen slides so that the result was safe and assertive that there was no residual tumor. When the paraffin blocks are prepared, the structures become more evident and clearer due to thinner histological sections, allowing a more detailed analysis. In 98% of the cases, the result of the intraoperative frozen section was in agreement with the final result of the anatomopathological examination of the surgical specimen (paraffin processing). According to the experience of the pathologists in the service, this interpretation disagreement, generating a false negative frozen section result, is commonly related to thick histological sections, inflammatory infiltrates, follicular proliferation, and trichilemmal lesions. A common diagnostic pitfall when freezing skin tumors is to mistake tangentially sectioned hair follicles for BCC. Inflammation may also be mistaken for a tumor or may obscure an underlying malignancy.^11^

The standard intraoperative freezing technique (“bread-loaf” technique) typically involves sections through specimens on their horizontal and longitudinal axes. According to the literature, approximately 44% of the entire margin is normally assessed, which partly explains why tumors initially reported as “completely excised” occasionally recur.^12^

**Figure 5:**
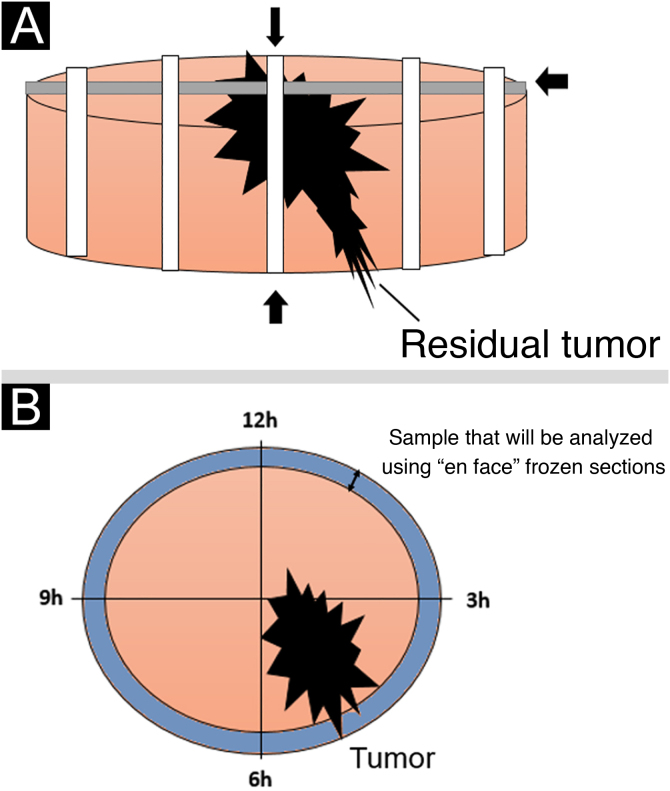
(A), Schematic drawing demonstrating the standard freezing technique: the sample is sectioned by removing transverse strips at right angles to each other. (B), The “*en face*” freezing technique is demonstrated, which examines all peripheral and deep margins of the lesion.

The intraoperative assessment of surgical margins using “*en face*” frozen section described in the present study offers certain benefits over standard freezing techniques. One of these benefits is that this technique examines all peripheral and deep margins, preventing false-negative results, in addition to being a fast and easily available technique.^5^, ^7^, ^8^, ^13^, ^14^, ^15^ Fig. 5 shows a schematic drawing of the “*en face*” freezing technique and the standard freezing technique.

However, some limitations of the “*en face*” freezing technique can be mentioned, such as it does not allow determining the distance between the tumor and the final margin of the specimen. Even if the margin is negative, the tumor may be just a few micrometers away. Thus, although a free clinical margin of 4 mm is not required, some degree of free clinical margin is still preferred when using the “*en face*” technique, aiming to minimize the risk of a small free histological margin.^7^, ^16^, ^17^ According to the literature, a large part of tumor recurrences occur after the fifth year of treatment. This finding emphasizes the importance of long-term follow-up.^2^ This fact demonstrates a drawback in the present study, as a median follow-up time of three years was attained. However, the data of the present are comparable to other studies in the literature on the use of intraoperative “*en face*” frozen sections.

Lesions that showed disagreement between the result of the intraoperative frozen section and the result of histopathological processing in paraffin blocks were submitted to complementary treatment (new surgical excision).

## Conclusion

The intraoperative “*en face*” frozen section technique is a fast, safe and reliable technique to ensure tumor-free surgical margins for BCCs and SCCs. The tumor recurrence rate in the present study is acceptable, according to the literature.

## Financial support

None declared.

## Authors' contributions

Ana Carolina Vasconcellos Guedes Otsuka: Collection of data, or analysis and interpretation of data; drafting and editing of the manuscript or critical review of important intellectual content; approval of the final version of the manuscript.

Eduardo Bertolli: Design and planning of the study, statistical analysis; drafting and editing of the manuscript or critical review of important intellectual content; effective participation in research orientation; approval of the final version of the manuscript.

Mariana Petaccia de Macedo: Design and planning of the study; effective participation in research orientation; approval of the final version of the manuscript.

Clovis Antonio Lopes Pinto: Effective participation in research orientation; approval of the final version of the manuscript.

João Pedreira Duprat Neto: Drafting and editing of the manuscript or critical review of important intellectual content; intellectual participation in the propaedeutic and/or therapeutic conduct of the studied cases; approval of the final version of the manuscript; effective participation in research orientation.

## Conflicts of interest

None declared.
